# External Dacryocystorhinostomy; Success Rate and Causes of Failure in Endoscopic and Pathologic Evaluations

**Published:** 2017-07-01

**Authors:** Hassan Ghasemi, Sajedeh Asghari Asl, Mohammad Ebrahim Yarmohammadi, Farhad Jafari, Pupak Izadi

**Affiliations:** 1 *Dept. of Ophthalmology, Medical School, Shahed University, Tehran, Iran*

**Keywords:** Lacrimal Sac, Nasolacrimal Duct, Epiphora, Dacryocystorhinostomy

## Abstract

**Background and Objectives:**

External dacryocystorhinostomy (DCR) is the method of choice to treat nasolacrimal duct (NLD) obstruction and the other approaches are compared with it, with a failure rate of 4% to 13%. The current study aimed to assess the causes of failure in external DCR by postoperative endoscopic and pathological evaluation.

**Methods:**

The current retrospective cross sectional study followed-up113 patients with external DCR and silicone intubation for three months. Silicone tubes were removed after the third months. Failure was confirmed based on the clinical findings and irrigation test. Paranasal sinus computed tomography (CT) scanning, and endoscopic and pathological evaluations were performed in the failed cases.

**Results:**

Totally, 113 patients underwent external DCR. The patients included 71 females and 42 males. The mean age of the patients was 55.91 years; ranged from 18 to 86. Epiphora was the most common complaint before surgery (90.3%). Clinically, epiphora continued in 17 cases (15%), of which 94.11% had at least one sinus CT abnormality and 82.35% had at least one endoscopic abnormality. The most common endoscopic findings were deviated septum (70.6%), scar tissue (52.94%), concha bullosa (46.9%), septal adhesion (47.05%), enlarged middle turbinate (41.2%), and sump syndrome (11.7%). The failure was significantly associated with the chronicity of the initial symptoms (P-value=0.00). Pathologically, there were significant relationship amongst the failure rate, scar formation, and allergic rhinitis (P-values =0.00 and <0.05, respectively).

**Conclusion:**

Preoperative endonasal evaluation and consultation with an otolaryngologist can improve surgical outcomes and help to have a better conscious to intranasal abnormalities before external DCR surgery.

## Introduction

The lacrimal system is a borderland of the disciplines of ophthalmology and otorhinolaryngology, which cooperate in the clinical setting to treat and cure the disorders of this system. The history of the lacrimal system disorders and nasolacrimal duct (NLD) obstruction studies date back to 2200 B.C. (Hammurabi era). Since then, several therapeutic strategies are proposed to eliminate tearing and obstruction (1). Primary NLD obstruction is one of the problems that afflict the humans from the past and with this disorder are a significant part of the patients referred to ophthalmology clinics. NLD obstruction often presents irritative symptoms such as tearing, pain, burning, redness, and swelling of the eyes (2), which can be due to different causes, including congenital, traumatic, iatrogenic, lithiasis, and infections (3). Primary acquired nasolacrimal duct obstruction (PANDO) is the most common cause of obstruction in adults, which often occur more in females in the fifth and sixth decades of life. The incidence of obstruction is less at both ends of the age spectrum due to less secretion of tears (3,4). Tearing is the main complaints of patients that occurs due to an obstruction in tear drainage system (5). Epiphora and dacryocystitis are the annoying symptoms that result from lacrimal duct obstruction in about 1% of adults and 3% to 5%of children (6). Obstruction can occur anywhere in the lacrimal system, but the most common site of obstruction is at the junction of the lacrimal sac and NLD (7). Dacryocystorhinostomy (DCR) is the procedure of choice to treat NLD obstruction by creating anostium for bypassing tears into the nasal cavity. There are two main approaches for this surgery: the conventional (external) and intranasal (endonasal) (3,8). External DCR is the most common surgery and the preferred method among ophthalmologists. It is performed by standard skin incision and removal of maxillary and lacrimal bones to create a connection path between the lacrimal sac and nasal cavity mucosa (9). It is the gold standard treatment of PANDO and other methods are measured and compared with it. This method was first introduced in 1904 by Adeo Totti (10, 11).

The success rate of this approach varies in different studies from 63% to 97%. Overall, there is still a failure rate of4% to13%in which the patients' epiphora recurs (3,12). The main causes of the failure of this method were evaluated in some studies. On the other hand, anatomical variations and intranasal pathologies are the most common reasons that can cause narrowing of the nasal airway and the subsequent failure of the surgery (1). Some causes of the failure include granulation of tissue and scar formation, insufficient rhinostomy, presence of nasal polyps and rhinosinusitis, inappropriate location or closure of the ostium, concha bullosa, intranasal adhesion, abnormal size of fistula, sump syndrome, previous maxillofacial trauma, enlargement of aggernasi cells, and paradoxical or hypertrophic middle turbinate (12-21). 

Therefore, intranasal evaluation and the ears, nose and throat (ENT) consultation and/or diagnostic nasal endoscopy can discover the intranasal pathologies that may lead to the failure of DCR (22). Therefore, the current study aimed at evaluating the patients who underwent external DCR and had recurrent tearing by diagnostic paranasal sinus computed tomography (CT) scan before and during endoscopic revision.

## Material and Methods

This retrospective, cross sectional study was conducted in Mustafa Khomeini Hospital, Tehran, Iran, from 2005 to 2013. The study protocol was approved by the Ethics Committee of Shahed University, Tehran, Iran, according to the Helsinki declaration. Additionally, informed consent was obtained from all eligible participants.

A total of 113 patients with positive regurgitation test from both canaliculi underwent external DCR with silicon intubation by the same surgeon. None of them had canalicular obstruction. They were followed-up for three months and evaluated after silicon removal for recurrence of symptoms. Demographic information, initial symptoms, any complications during and after surgery, signs of recurrence or failure and outcomes of each patient were collected from available medical charts and recorded in the datasheets. Patients were evaluated in the first and third days, and first week after surgery for short-term complications; then, they were additionally followed-up for three months. Silicone tube was removed at the third months after surgery. Patient with tearing or purulent discharge just after or a few days after silicone tube removal were considered as the failure. Besides the symptoms, failure was confirmed by negative irrigation test. The patients who fulfilled the criteria were included in the study. The inclusion criteria were the recurrence of symptoms during the three-month follow-up and being a candidate for diagnostic endoscopic revision. To consider revision endoscopic evaluation, paranasal sinus CT scans were conducted in the patients with failure of the surgery. In the treatment progress, failed cases underwent endoscopic revision surgery with 0 and 30 degrees endoscope (Stroze, Germany). Any clinically visible pouch such as lacrimal sac remnants with fluid retaining capacity, confirmed by a 30 degree endoscopic lens were considered as sump syndrome. Pathologic specimens were obtained from the site of obstruction and their recorded endoscopic and pathologic findings were reviewed.

All information including demographic data, results of CT scans, endoscopic findings, and surgical outcomes were recorded, and the data were analyzed using SPSS version 21.

## Results

Totally, 113 patients underwent external DCR including 71(62.8%) female and 42 (37.2%) males. The age range of the patients was 18 to86 years (mean: 55.91 years). Mean onset of symptoms was six years. The most primary complaint of patients before surgery was epiphora (102 patients, 90.3%). None of the initial 113 cases and the failed cases had canalicular obstruction. Also three cases showed functional obstruction that two of them had clinical sump syndrome.

In the patients with failure of surgery (17), 94.11%had at least one sinus CT abnormality. The most common paranasal sinus CT scan finding was septal deviation (76.47%), followed by rhinitis (35.3%) and concha bullosa (29.4%).The most common endoscopic finding was septal deviation (70.6%), followed by septal adhesion (47.05%) and enlarged middle turbinate (41.2%).Also, remnant of bottom of lacrimal sac or sump syndrome was (11.76%) (Tables 1 and 2). 

In failed cases, 82.35% had at least one endoscopic abnormality. There were several concurrent endoscopic findings in 12 failed cases (70.58%). Most of the concurrent findings included septal deviation with enlarged middle turbinate and concha bullosa. Most of the abnormal pathologic findings were scar tissue formations at the site of incision (nine patients, 52.94%), and only two patients (11.76%) had no pathologic findings (Table 3).

**Fig 1:**
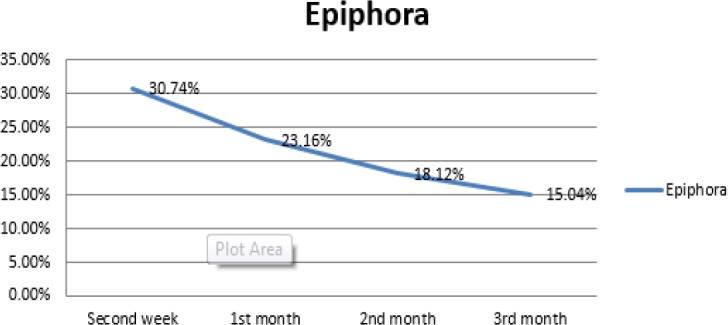
Clinical Success Rate and Distribution of Tearing at Follow-up after the Surgery

During the three-month follow-up, 38 (33.62%) patients still had tearing in the first two weeks after the surgery, but this symptom gradually decreased to 17 (15.4%) patients at the end of the three-month follow-up. As mentioned earlier, patients with tearing up to three months after the surgery, with negative irrigation test results, were considered as the failure. Anatomical success rate of surgery was87.6% (Fig 1).

**Table 1:** ParanasalSinus CT Scan Findings in patients with failure of surgery

Findings	Number	Percent
Septal deviation	13	76.47
Rhinitis	6	35.3
Concha bullosa	5	29.4
Enlargement of bullaethmoidalis	5	29.4
Aggernasi cells	4	23.5
Sinusitis	4	23.5
Elongated uncinate process	2	11.8

The most frequent paranasal sinus CT scan findings in the patients with failure of surgery were septal deviation, followed by rhinitis and concha bullosa.

**Table 2:** Endoscopic Findings in the patients With Failure of the Surgery

Endoscopic findings	Number	Percent
Septal deviation	12	70.6
Septal adhesions	8	47.05
Enlargement of middle turbinate	7	41.2
Fibrosis	4	23.52
Ostial stenosis	3	17.64
Sump syndrome	2	11.76
Lower adhesion	2	11.76
Uncinate process	1	5.8

The most frequent endoscopic findings in the patients with failure of surgery were septal deviation followed by septal adhesion and enlargement of middle turbinate.

**Table 3:** Distribution of Pathologic Findings in patients with failure of surgery

Pathologic Findings	Number	Percent
Scar tissue	9	52.94
Chronic inflammation	7	41.17
Granulation tissue	6	35.29
Polyp	2	11.76
No findings	2	11.76

The most frequent pathologic findings in the patients with failure of surgery were scar tissue, followed by chronic inflammation and granulation tissue.

Gradual decrease in epiphora during three months after operation

Apart from the three cases with functional obstruction, clinical success rate was84.95%. Failure rate was higher in females than in males, but the difference was insignificant. The failure rate was significantly associated with the chronicity of the initial symptoms (P-value= 0.00). There were significant relationships among failure rate, deep scar formation, and allergic rhinitis (P-values =0.00 and <0.05, respectively).

## Discussion

External DCR is an effective treatment to relieve the symptoms in primary acquired NLD obstruction. Although numerous percentages of failure rates and relapse of symptoms were reported in different studies, the success rate of this method is reported from 75% to 97% (4). The current study assessed the success rate of a group of patients who underwent external DCR surgery and, then, the failed cases were re-evaluated by CT scan and revision endoscopic DCR for possible factors involved in the surgical failure. The patients with tearing and negative irrigation test three months after the surgery were considered as the failure of the surgery. The rate of clinical epiphora after silicone tube removal, three months after the surgery was 15.4% (success rate: 84.6%). Ben Simon et al., reported a success rate of 76.7% in 176 external DCR surgeries (13). Eyigor H et al., reported that 73.3% of patients with NLD obstruction had at least one or more paranasal sinus CT abnormalities. They found a positive correlation between sinonasal abnormalities and NLD obstruction (23). The current study found 94.11% CT abnormalities before endoscopic revision surgery in the failed cases. Many cases with abnormalities could change the method of surgical intervention. Therefore, an intranasal evaluation, ENT consultation, and if necessary, and obtaining a paranasalsinus CT before any intervention are extremely important (24).

In the external method, the surgeon opens the lacrimal bone from the lateral aspect of the nasal bone and creates an ostium between the medial wall of sac and nasal mucosa. Occasionally, due to poor accessibility, the medial wall and bottom of the lacrimal sac may not sufficiently open into the nasal cavity and therefore, a pouch remnant of the lacrimal sac may remain that is called sump syndrome. This may cause fluid accumulation in the lower part of the lacrimal sac. Subsequently, accumulation of tears and frequent infections may gradually close the ostium by mucins clots and inflammations (16).

In revision of the endoscopic evaluation of the patients with failure of surgery, unintentionally remained inferior part of the lacrimal sac and accumulation of gravity dependent fluids, best visualized by a 30 degree endoscopic lens, were considered as sump syndrome. This finding was in agreement with those of the other studies (13; 25).Septal deviation, septal adhesion, and other intranasal abnormalities play important roles in increasing failure rate via the induced inflammation. Elmors yet al., in 65 failed cases reported intranasal adhesions (30%), septal deviation, concha bullosa and abnormal size fistula (each one 12%), rhinitis (9%), contact granuloma (9%), sump syndrome (5%), closed ostium (3%), and functional failure (15%) (15). The current study showed more intranasal abnormalities and less functional failure that may be due to the community differences and variations. The most frequent pathologic findings at the site of closed ostium were scar tissue, followed by chronic inflammation, granulation tissue, and polyp in some of the patients. Pathologic preparation of tissue is not a routine investigation, but the presence of scar tissues by clinical estimation were verified by few authors (9).

The most important limitation of the current cross sectional study was lack of a control group. Therefore, direct relationships and assignments of the failure to anatomic abnormalities/variations are limited.

## Conclusion

Based on the findings of the present study, it is suggested that intranasal pathologies are amongst the most common causes of failure in external DCR. Preoperative endonasal evaluation and otolaryngologist consultation, with or without paranasalsinus CT scans, can discover the intranasal abnormalities, and increase the success rate of surgery.
